# Reactivated CMV Proctitis/Anitis Presenting as a Localized Proximal Anal Swelling and Anal Pain in a Diabetic Patient: Case Report and Literature Review

**DOI:** 10.3390/v17081023

**Published:** 2025-07-22

**Authors:** Dua Abuquteish, Ayat Al Oqaily, Lama Bataineh, Bashar Khater

**Affiliations:** 1 Department of Microbiology, Pathology and Forensic Medicine, Faculty of Medicine, The Hashemite University, Zarqa 13133, Jordan; 2 Department of Pathology and Laboratory Medicine, King Hussein Cancer Center, Amman 11941, Jordan; 3 Department of Pathology and Laboratory Medicine, King Abdullah University Hospital (KAUH), Jordan University of Science and Technology (JUST), Irbid 22110, Jordan; analoqaily@just.edu.jo (A.A.O.); lolo.bat34@gmail.com (L.B.); 4 Faculty of Medicine, The Hashemite University, Zarqa 13133, Jordan; basharkhater01@gmail.com

**Keywords:** CMV, reactivated, proctocolitis, anal pain, immunocompetent, diabetes

## Abstract

Background: Cytomegalovirus (CMV) colitis is commonly seen in patients who are immunodeficient or have inflammatory bowel disease. Among the gastrointestinal sites affected by CMV, the colon is the most frequently affected, though rectal involvement is relatively rare. Reactivated CMV proctitis primarily occurs in elderly patients with comorbidities and is quite uncommon in immunocompetent individuals. Patients with reactivated CMV typically present with symptoms such as diarrhea, hematochezia, or tenesmus. Case presentation: We report a case of a female patient with uncontrolled diabetes who presented to the clinic complaining of perianal pain. She had no history of diarrhea or rectal bleeding. Lower GI endoscopy reported a small, localized, approximately 0.5 cm swelling in the proximal anal canal in addition to sigmoid diverticulosis. The biopsy revealed a small ulcer at the anorectal junction caused by CMV and confirmed by immunohistochemistry. Unfortunately, the patient was lost to follow-up before antiviral therapy could be initiated. Conclusions: This case highlights an uncommon presentation of reactivated CMV proctitis in an older diabetic patient presenting solely with perianal pain. Clinicians should maintain a high index of suspicion for CMV infection in elderly patients with comorbidities, even when classical colitis symptoms are absent, to avoid delayed diagnosis and management.

## 1. Introduction

Cytomegalovirus (CMV) is a double-stranded DNA virus and is part of the herpesvirus family. It usually affects immunocompromised patients, may cause severe systemic disease, and negatively affects clinical outcomes. It usually results in a self-limited disease in the healthy population [1].

Human CMV is a member of the Betaherpesvirinae subfamily and has the largest genome among the known DNA viruses, encoding over 170 proteins. The virus consists of three layers: an outer lipid envelope, a tegument, and an envelope studded with glycoproteins. The functions of the herpesvirus capsid are mainly packaging, transporting, and delivering the viral genome. Upon binding to host cell receptors through viral glycoproteins, the nucleocapsid enters host cells. This can occur via fusion between the cell membrane and the viral envelope, allowing the capsid to be transported to the nucleus, where it initiates viral replication. The tegument protein pp71 plays a critical role by promoting the degradation of the cellular repressor Daxx, which derepresses viral immediate–early (IE) gene expression and triggers the lytic cycle. Within hours of infection, IE genes, such as IE1 and IE2, are expressed and are crucial for the subsequent expression of early (E) and late (L) genes. E genes, which are expressed prior to viral DNA replication, encode proteins necessary for viral genome replication and the regulation of host cell functions. In contrast, L genes are expressed after the onset of viral DNA replication and primarily encode structural proteins required for the assembly of new virions [2,3].

Three CMV infection patterns were described: invasive tissue disease, particularly in immunocompromised patients, and latent infection and mononucleosis-like syndrome, which are more frequently observed in immunocompetent patients. The gastrointestinal tract is commonly involved in tissue-invasive CMV disease, reported in 30% of cases [4], with the colon being most frequently affected, followed by the esophagus [5]. However, rectal involvement is rare. CMV proctitis can manifest in two forms: primary and reactivated. Reactivated CMV proctitis occurs primarily in elderly patients with comorbidities, mainly those with diabetes, and it is uncommon in immunocompetent patients. In the literature, patients with reactivated CMV mainly presented with diarrhea, hematochezia, or tenesmus during hospitalization.

Clinical presentation, imaging, and endoscopy findings are usually non-specific and could be present in other etiologies, such as inflammatory bowel disease and ischemic and infectious colitides [6]. Patients with CMV colitis documented various clinical presentations, such as diarrhea, rectal bleeding, abdominal pain, weight loss, fever, and colonic perforation [7,8]; diarrhea and rectal bleeding were encountered most frequently [8].

Herein, we present a case of a female patient with uncontrolled diabetes who presented to the clinic complaining of perianal pain. Lower GI endoscopy reported the presence of a small, localized swelling in the proximal anal canal in addition to sigmoid diverticulosis. The biopsy of the swelling confirmed the diagnosis of CMV proctitis/anitis by histology and CMV immunohistochemistry. The patient did not come back to the clinic. Hence, follow-up was not conducted.

Nevertheless, our patient’s clinical picture was vague and considered uncommon for CMV proctitis. Therefore, we report this case to increase the awareness of possible presentations of reactive CMV proctitis, particularly in people with diabetes mellitus. Clinicians always have a high suspicion of CMV in immunodeficient patients, but this is not the case for immunocompetent patients, with or without comorbidities.

## 2. Materials and Methods

### Case Presentation

This case involves a 58-year-old female patient with diabetes mellitus (uncontrolled), hypertension, left eye blindness, and glaucoma who was on several medications, such as brimonidine, latanoprost, dorzolamide, and midazolam. She presented to the gastrointestinal outpatient clinic complaining of perianal pain. The patient denied a history of fever, diarrhea, or rectal bleeding. Laboratory investigations included CBC (WBC of 6.5 × 10^9^/L, absolute lymphocyte count of 2.2 × 10^3^/µL, hemoglobin 13.3 g/dL, and platelets 275 × 10^9^/L), a random HbA_1_c of 9.6%, and a CRP of 18.3 mg/dL. She has no known history of malignancy, HIV infection, organ transplantation, or use of immunosuppressive medications. Her family history is only significant for diabetes; her dad and two aunts have type 2 diabetes. She does not smoke or drink alcohol. She is a retired teacher. She has no history of recent travel. Upon physical examination, she was afebrile with stable vital signs. Her abdominal examination was normal, with no tenderness, distention, or organomegaly. The last documented CMV serology was performed during the previous clinic visit, two months prior to her presentation. It showed CMV IgG at 3.1 U/mL and IgM at 2 AU/mL.

**Figure 1:**
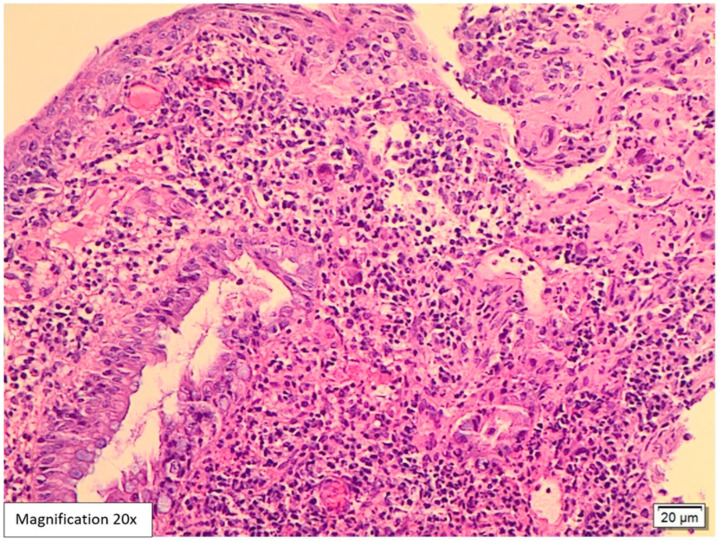
Hematoxylin and eosin (H&E)-stained biopsy from the swelling at the anorectal junction, 20× magnification. This fragment is lined by squamous and colonic mucosa with focal ulceration, crypt architecture distortion, cryptitis, and crypt abscess formation. The lamina propria is expanded by mixed neutrophilic and lymphoplasmacytic infiltrates. The scattered large atypical stromal cells containing intranuclear basophilic inclusions are identified among the inflammation.

**Figure 2:**
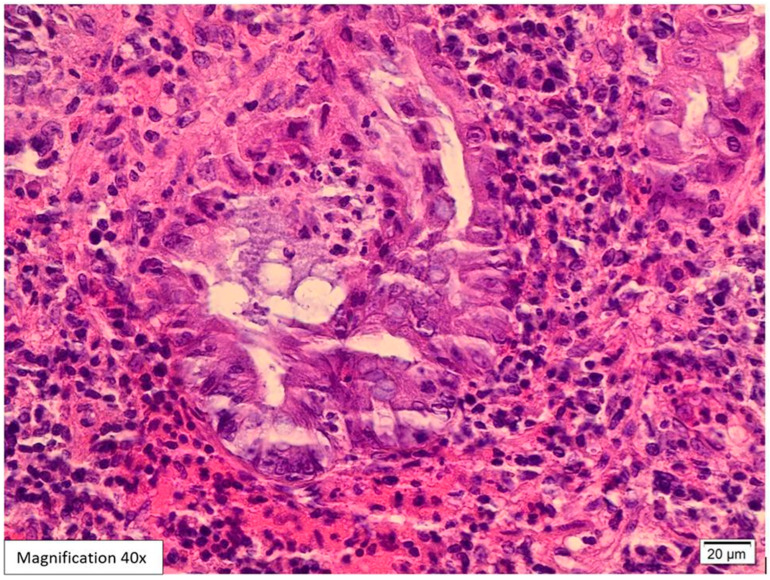
H&E-stained biopsy from the swelling at the anorectal junction, 40× magnification. The higher magnification (40×) highlights the cryptitis and crypt abscess surrounded by inflamed lamina propria.

**Figure 3:**
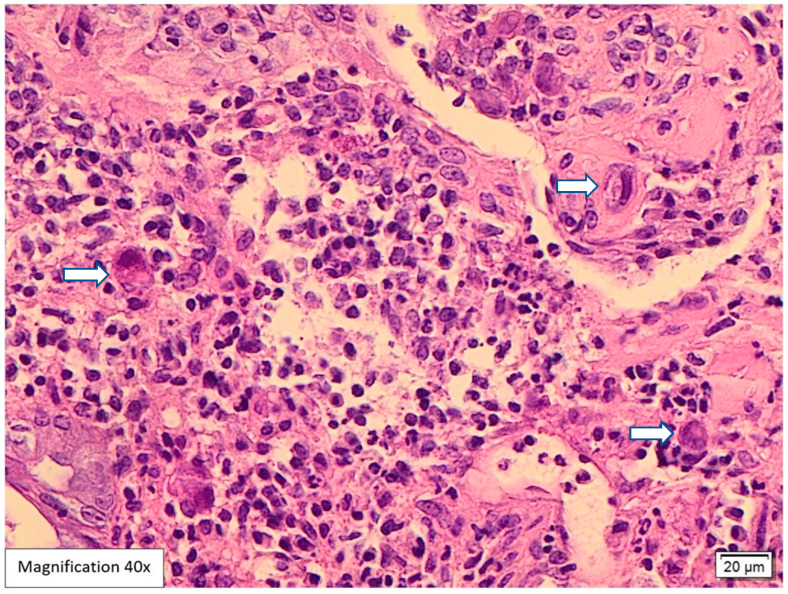
H&E-stained CMV inclusions, 40× magnification. The higher magnification (40×) highlights CMV inclusions with atypical stromal cells containing intranuclear and intracytoplasmic basophilic inclusions (white arrows).

**Figure 4:**
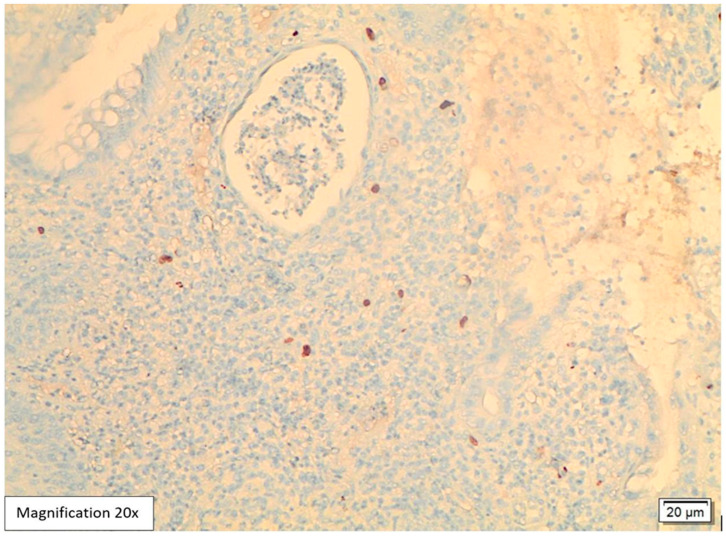
Positive CMV immunohistochemistry, 20× magnification. CMV immunohistochemistry is positive in the atypical scattered stromal cells, confirming the diagnosis.

Lower GI endoscopy showed a small, localized, approximately 0.5 cm swelling in the proximal anal canal in addition to sigmoid diverticulosis. The rest of the colonic mucosa was unremarkable. A biopsy was performed and sent to the pathology lab. The attached endoscopic report did not include a possible etiology for the anal swelling. Histological examination of the hematoxylin and eosin slides (H&E) showed five fragments lined by colonic and squamous mucosa with focal ulcerations and mild distortion in the crypt architecture. The lamina propria revealed intense acute inflammatory cell infiltrates, cryptitis, crypt abscess formation, and mixed lymphoplasmacytic infiltrates (Figure 1 and Figure 2). Among the inflammation, there were large scattered atypical stromal cells containing intranuclear and intracytoplasmic basophilic inclusions (Figure 3). CMV immunostaining was positive in these cells, confirming the diagnosis of CMV proctitis/anitis (Figure 4). No other viral inclusions or infectious microorganisms were present, and there were no granulomas, dysplasia, or malignancy.

After a histological diagnosis of CMV proctitis was made, the patient did not return for follow-up or antiviral treatment, and no further information regarding her clinical course was available.

Consent section: Informed consent was obtained from the patient to publish this case report and the accompanying images.

## 3. Discussion

CMV colitis frequently occurs in immunodeficient patients, including patients with human immunodeficiency virus (HIV) infection, post-transplant patients, and patients with a history of malignancy or chemotherapy [1]. In addition, CMV colitis is common in patients with inflammatory bowel disease, with a prevalence ranging from 1.5 to 4.5%, and is more commonly observed in patients with ulcerative colitis than those with Crohn’s disease [9,10]. Although CMV colitis is uncommon in immunocompetent patients, several factors may increase the risk of infection [8]. Such factors include rheumatological, neurological, and renal diseases, ICU admissions, and exposure to certain medications and steroids.

Clinical presentation, imaging, and endoscopy findings are usually non-specific for CMV colitis. Various clinical presentations have been documented, such as diarrhea, rectal bleeding, abdominal pain, weight loss, fever, and colonic perforation [7,8], with diarrhea and rectal bleeding more frequently present [8]. In addition, other rare presentations may mimic malignancy [11]. For instance, Cohen et al. [12] reported a case of rectal bleeding and the sensation of a mass protruding through the anus. Also, a recent abstract [13] described CMV infection as a firm rectal mass in an HIV patient.

CMV colitis has no specific appearance on endoscopy. Nonetheless, the only significant documented feature is well-defined, punched-out ulcers [14,15]. Other described patterns included ulcerations and a cobblestone-like appearance, pseudomembranes or ischemic colitis, and toxic megacolon [16,17,18]. In a study performed to assess the endoscopic findings in nine immunosuppressed patients with CMV colitis [6], colonic mucosal inflammation, ulceration, and erythema were the most pronounced findings in lower GI endoscopy, and the sigmoid colon was most affected. CT scan imaging showed that all nine patients had pericolonic stranding and bowel wall thickening. In addition, the most common presenting symptoms were bloody stools, abdominal pain, and diarrhea. They concluded that imaging and endoscopy might generate similar results for several forms of colitides, such as ischemic, inflammatory, and infectious.

The most affected site for CMV colitis has been reported differently in the literature. Some found CMV lesions to be distributed evenly in the colon [19]; others noted that the sigmoid colon is the preferable site for CMV colitis in immunocompetent patients [20]. However, rectal CMV involvement appears to be uncommon. Primary CMV proctitis occurs in young patients after unprotected anal intercourse as a mononucleosis-like illness associated with rectal bleeding. On the other hand, reactivated CMV proctitis usually occurs in elderly patients with comorbidities, such as IBD, DM, and multiorgan failure, and no history of anal intercourse [21].

Bromley et al. (2024) [22] conducted a recent systemic review to assess the pathophysiology of CMV proctocolitis in immunocompetent adults and to explore the associated factors, clinical presentations, and endoscopic findings in these patients. Eight manuscripts were included in their review. They found that cardiovascular, renal, autoimmune, and DM diseases contributed to CMV reactivation in the colon and rectum. Other factors included older age, low body mass index, receptive anal sex, infectious factors (urinary tract infections, shigellosis, COVID-19, hepatitis C, and sepsis), and hospitalization.

DM was one of several medical conditions associated with CMV proctocolitis in immunocompetent patients, highlighting that even without classic immunosuppression, DM may increase the risk of tissue-invasive CMV disease.

Reported symptoms included rectal bleeding, diarrhea and melena, fever, nausea/vomiting, abdominal pain/bloating, and constipation. Lower endoscopy showed ulcers, erosions, erythema, colitis (including pseudomembranous), and mass-like lesions.

Limitations for their review were the small number of manuscripts and the small number of participants.

Lee, Chen and Lu, 2017 [23] documented a case of reactivated CMV proctitis in an immunocompetent patient and performed a literature review for similar cases of reactivated CMV proctitis. Their search yielded eleven patients from five published papers with cases limited to the rectum. Most of them were elderly patients or people with diabetes mellitus. They illustrated that these patients might be more vulnerable to CMV proctitis, which is sometimes associated with CMV colitis. Nevertheless, 73% of these cases occurred as nosocomial diarrhea preceded by an acute life-threatening condition during hospitalization. The main presenting symptoms were diarrhea, rectal bleeding, and tenesmus. They concluded that reactivated CMV proctitis in immunocompetent patients is rare and reported mainly in elderly patients with comorbidities, especially DM [23].

CMV proctitis and colitis usually occur concomitantly. However, studies showed that immunocompetent patients with immunity disturbances such as DM might be more susceptible to CMV proctitis and/or colitis than to colitis alone [19,23,24].

When diagnosing CMV colitis, symptoms of CMV viremia, such as pharyngitis, splenomegaly, and lymphadenopathy, are usually absent in these patients [1]. Additionally, approximately 40% of the adult population is seropositive for CMV; hence, blood serology has no value in the diagnostic workup [25]. IgG and IgM tests showed no diagnostic value. Although CMV IgM testing can detect acute CMV systemic infection or acute systemic reactivation, it does not help in diagnosing CMV colitis [5]. Even in patients with IBD, serology had limited utility in diagnosing CMV reactivation [26]. Sensitivity for detecting CMV tissue involvement varied widely, from 15% to 60% in IBD patients from a 2004 cohort of 64 IBD patients, which included 10 patients with CMV infection [27,28]. Previously, viral culture was considered the gold standard for detecting CMV with high specificity and sensitivity. However, it takes one to three weeks to receive the results, which might cause a delay in diagnosis and treatment. Thus, a definite diagnosis is made from histology and immunohistochemistry (IHC) and is now the gold standard for diagnosis [29]. The H&E-stained slides showed 82% sensitivity, increasing to 96% with IHC [30]. PCR is sensitive and specific in CMV detection in formalin-fixed paraffin-embedded blocks; therefore, it is suggested in cases where the CMV IHC is indeterminate and there is high clinical suspicion of CMV infection [25].

Our patient tested positive for CMV IgG and negative for CMV IgM two months prior to the current presentation. This indicates a previous exposure and a latent infection. The patient also exhibited localized biopsy-proven disease without systemic signs associated with primary CMV infection, suggesting that this is a reactivation of latent CMV rather than a new primary infection.

CMV usually infects endothelial cells and mesenchymal cells [31]. In histological examinations, these cells are usually 2 to 4 times larger compared to normal cells and harbor intranuclear basophilic inclusions (Cowdry bodies). If a clear halo surrounds inclusions, it gives the appearance of an “Owl’s eye” [29,31,32]. These cells also reveal thick nuclear membranes and coarse red intracytoplasmic granules [29]. In addition, the background colonic mucosa can show features mimicking IBD, such as crypt architectural distortion, cryptitis, crypt abscess, increased apoptotic bodies, basal lymphoplasmacytic infiltrates, and mucin depletion, along with fissuring ulcers and pseudopolyps [33]. Vasculitis and thrombus formation in microvessels can also be found [32].

In our case, histological examination for the anal swelling biopsy revealed typical histological appearances for CMV infection. The H&E slides showed fragments lined by colonic and squamous mucosa with focal ulcerations, mild crypt architecture distortion, cryptitis, and crypt abscess formation (Figure 1 and Figure 2). The lamina propria was expanded by mixed neutrophilic and lymphoplasmacytic infiltrates. Among the inflammation, scattered large atypical stromal cells containing intranuclear basophilic inclusions were identified; some were also surrounded by a clear halo “Owl’s eye” (Figure 3). Therefore, we ordered CMV immunohistochemistry, which was positive in these cells, confirming the diagnosis of CMV proctitis/anitis (Figure 4). There was no histological evidence of other viral inclusions, infectious microorganisms, granulomas, dysplasia, or malignancy.

CMV colitis is life-threatening, mostly in immunocompromised patients [34]. On the contrary, it is self-limited in immunocompetent young patients with no comorbidities [19]. Nonetheless, the age of 55 and above and being a male are considered negative prognostic factors [35]. Other reported factors that adversely affect the outcome and require treatment are chronic renal diseases, diabetes, and non-hematological malignancies [36].

Compared to non-diabetics, infectious diseases are more frequent in people with diabetes mellitus, which is attributed to hyperglycemia causing neutrophil function impairment and depression of humoral immunity. Moreover, microangiopathies, urinary and gastrointestinal dysmotility, and increased medical intervention play a role. Some infections are seen only in people with diabetes mellitus, including rhinocerebral mucormycosis, gangrenous cholecystitis, and malignant external otitis [37]. Additionally, people with diabetes showed an increased risk of gastrointestinal tract infections, such as oral and esophageal candidiasis, helicobacter pylori infections, and emphysematous cholecystitis [38,39,40,41]. There have also been studies that demonstrated associations between diabetes and viruses such as Hepatitis C, Hepatitis B, and Enteroviruses [42,43,44,45].

Treatments inhibiting CMV fall into two main categories: those that directly target viral components and those that disrupt host cell functions. Virus-targeted inhibitors work by interfering with viral replication and inhibiting key proteins, such as viral DNA polymerase. Among these, nucleoside and nucleotide analogs and ganciclovir (and its oral prodrug, valganciclovir) are considered first-line treatments for human CMV infections. These agents require activation by the CMV UL97 kinase and cellular enzymes to inhibit viral DNA polymerase, resulting in DNA chain termination. However, they often result in bone marrow suppression. Second-line options include cidofovir, which also inhibits DNA polymerase, and foscarnet, an inorganic pyrophosphate analog that blocks the viral polymerase without requiring intracellular activation. Additionally, recent agents, such as letermovir, a viral terminase complex inhibitor, prevent viral DNA cleavage and packaging, while maribavir specifically inhibits the HCMV UL97 kinase, thereby affecting DNA synthesis and viral egress. Maribavir has recently been approved for treating post-transplant HCMV infections. On the other hand, host-targeted antivirals target different aspects of host cell functions. While they vary in efficacy, these inhibitors share the advantage of not having viral resistance. An example of this type of antiviral is artesunate, which inhibits cellular factors such as Sp1 and NF-κB. Artesunate has shown promise in preclinical and some clinical settings without evidence of viral resistance [2].

In immunocompetent patients, there are no standardized guidelines on antiviral therapy, and treatment decisions should be individualized. Medications like ganciclovir can have side effects, such as bone marrow suppression, liver and kidney toxicity, and neurological effects, without proven significant benefits in outcomes. However, treatment may be appropriate for older males over 55 with severe disease and conditions that compromise their immune system, such as diabetes or chronic kidney disease. In patients with inflammatory bowel disease, especially those unresponsive to steroids, CMV reactivation does not always warrant antivirals unless tissues show high CMV levels or large ulcers are present [5]. Antiviral therapy may improve outcomes in severe cases; however, diagnosis should rely on both histology and IHC, as PCR alone may indicate viral presence without clinical relevance. Generally, therapy is considered for patients with severe illnesses, complications, or significant comorbidities, regardless of their immune status [46,47].

Furthermore, in a previous study, Lawlor and Moss (2010) [48] investigated whether CMV acts as a true pathogen or a bystander in patients with IBD, especially those with ulcerative colitis. CMV is rarely involved in mild cases but appears in up to 40% of severe, steroid-refractory UC cases. Detection by PCR may reflect incidental reactivation, especially without histologic confirmation. Reactivation is more common in immunosuppressed patients but does not always worsen outcomes or require treatment. Antivirals like ganciclovir may help some patients with confirmed CMV colitis, though evidence is limited. Current guidelines recommend treatment only when tissue involvement is confirmed, as CMV often represents a secondary phenomenon.

However, in the case of our patient, whose presentation was mild and localized, the clinician did not recommend antiviral therapy, advising only follow-up instead.

Our patient is diabetic with uncontrolled blood sugar and is older than 55. One of her listed medications is midazolam, which is reported to inhibit some functional aspects of the immune system [49]. Based on that, she could be considered mildly immunocompromised. However, some studies considered people with diabetes mellitus immunocompetent, as they are not as profoundly immunocompromised as HIV patients, transplant recipients, or patients on immunosuppressive therapies [23].

Our patient’s clinical picture with anal pain and endoscopic localized anal swelling, which appeared to be a small, localized ulcer caused by CMV, is a very uncommon presentation for reactivated CMV proctitis and is rarely reported. Therefore, the diagnosis relied mainly on histology and immunohistochemistry.

We believe that more studies are needed to highlight the importance of including CMV proctitis/proctocolitis in the differential diagnosis of people with diabetes mellitus presenting with common or uncommon gastrointestinal manifestations. Most studies in the literature describe CMV in people with diabetes mellitus who are debilitated and/or hospitalized.

Our findings highlight the importance of including CMV infections in differential diagnoses for patients older than 55 with comorbidities such as diabetes, especially if uncontrolled. In addition, keep in mind that the clinical picture of reactivated CMV can sometimes be vague and not limited to common CMV proctocolitis presentations such as diarrhea and rectal bleeding.

Physicians should be aware of such symptoms and carefully interpret them to avoid missing potential diagnoses. This case report supports the literature that elderly patients with comorbidities including, but not limited to, diabetes mellitus appear to be more prone to reactivated CMV proctitis.

## Data Availability

Data are contained within the article.

## Abbreviations

The following abbreviations are used in this manuscript:

CMV	Cytomegalovirus
H&E	Hematoxylin and eosin
IHC	Immunohistochemistry
CT	Computed tomography
IBD	Inflammatory bowel disease
COVID-19	Coronavirus disease 2019
DM	Diabetes mellitus
PCR	Polymerase chain reaction
ICU	Intensive care unit
